# Conducting Polymer-Based Nanohybrid Transducers: A Potential Route to High Sensitivity and Selectivity Sensors

**DOI:** 10.3390/s140203604

**Published:** 2014-02-20

**Authors:** Seon Joo Park, Oh Seok Kwon, Ji Eun Lee, Jyongsik Jang, Hyeonseok Yoon

**Affiliations:** 1 School of Chemical and Biological Engineering, Seoul National University, Seoul 151-742, Korea; E-Mails: seonjoopark86@gmail.com (S.J.P.); ohseok.kwon@yale.edu (O.S.K.); 2 Department of Chemical and Environmental Engineering, School of Engineering and Applied Science, Yale University, New Haven, CT 06511, USA; 3 Department of Polymer Engineering, Graduate School, Chonnam National University, Gwangju 500-757, Korea; E-Mail: alkinid@naver.com; 4 Alan G. MacDiarmid Energy Research Institute, School of Polymer Science and Engineering, College of Engineering, Chonnam National University, Gwangju 500-757, Korea

**Keywords:** conducting polymer, nanohybrids, transducers, chemical sensors, biosensors

## Abstract

The development of novel sensing materials provides good opportunities to realize previously unachievable sensor performance. In this review, conducting polymer-based nanohybrids are highlighted as innovative transducers for high-performance chemical and biological sensing devices. Synthetic strategies of the nanohybrids are categorized into four groups: (1) impregnation, followed by reduction; (2) concurrent redox reactions; (3) electrochemical deposition; (4) seeding approach. Nanocale hybridization of conducting polymers with inorganic components can lead to improved sorption, catalytic reaction and/or transport behavior of the material systems. The nanohybrids have thus been used to detect nerve agents, toxic gases, volatile organic compounds, glucose, dopamine, and DNA. Given further advances in nanohybrids synthesis, it is expected that sensor technology will also evolve, especially in terms of sensitivity and selectivity.

## 1. Introduction

Of organic materials, only conducting polymers have electrical and optical properties which are similar to those of inorganic semiconductors or sometimes metals. Thus, conducting polymers have been widely used to fabricate versatile chemical and biological sensors. Conducting polymers themselves are very sensitive to their surrounding environments, which makes them suitable for various sensor transducers. However, the sensitivity and selectivity of conducting polymer-based sensors still leave room for improvement. More specifically, there are several important factors which need to be improved for further success of conducting polymer-based sensors:

- sensitivity
- selectivity
- surface area
- environmental stability
- surface properties

The concept of using organic/inorganic hybrids as sensor transducers has been introduced to improve the important factors listed above [1–3]. There is a rich variety of possible combinations of organic-inorganic materials. Conducting polymers as the organic component can be also hybridized with one or more of the following materials:

- metal nanoparticles: gold, silver, platinum, silicon, palladium
- inorganic compounds: oxides, halides
- carbon nanomaterials: carbon nanotubes, fullerene, graphene

Polymers have traditionally held advantages over metal or inorganic materials, such as easy synthesis and processing, chemical and structural diversity, low weight, and flexibility [4]. Conducting polymers are a fascinating class of materials that combine the advantages of organic polymers with the electrical and optical properties of metal or inorganic semiconductors [5]. Accordingly, conducting polymers may be the best candidate for fabricating functional organic/inorganic hybrids. Moreover, conducting polymer-based hybrids are expected to have several synergistic properties between the polymer and the inorganic components, making them promising candidates for application in several fields such as catalysis, sensors, electronics, optoelectronics, and medicine [6,7]. On the other hand, nanostructured materials show unique properties depending on the size and shape [8,9]. In terms of sensor applications, moreover, they have beneficial advantages such as high surface area and small dimensions [10–12]. The enlarged surface area enhances the interactions between the materials and analytes, which leads to high sensitivity, and the small dimensions facilitate adsorption/desorption kinetics for analytes in the materials, which allows a rapid response time and high signal reproducibility [13]. In this review article, we focus on addressing synthesis strategies for obtaining conducting polymer-based nanohybrids and notable recent examples of sensor applications. In fact, many kinds of polymers including conducting polymers have been combined with carbon nanomaterials, such as fullerene, carbon nanotubes, graphene, and graphene oxide, to make diverse functional composites, because the synthesis routes and properties of the carbon nanomaterials have been well studied [14]. However, since there are already a couple of comprehensive review articles about carbon nanomaterials-based polymer composites, providing abundant information on the corresponding synthesis methods and applications, from a materials perspective, herein a considerable fraction will therefore be devoted to nanostructured hybrids that contain inorganic compounds such as metals or metal oxides/halides, and not carbon species.

## 2. Synthetic Routes to Conducting Polymer-Based Nanohybrids

A variety of methods have been used to prepare conducting polymer-based nanohybrids. A review article published in 2000 provides an excellent overview of conducting polymer nanocomposites [6]. Here, mainly recent research examples since 2000 will be discussed. The main synthesis strategies can be classified into the following four categories: (i) impregnation of metal precursors, followed by reduction; (ii) concurrent redox reactions; (iii) electrochemical deposition; (iv) seeding approach.

### (i) Impregnation, followed by reduction

**Figure 1.**
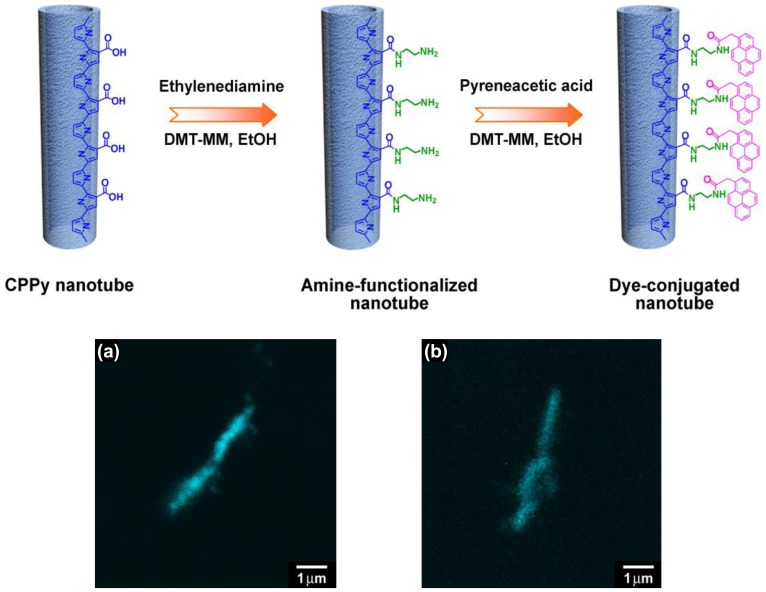
Schematic illustration of reaction steps for the covalent immobilization of fluorescent dye molecules on the surface of carboxylated PPy (CPPy) nanotubes: the first step involves the coupling reaction of CPPy nanotubes with ethylenediamine as a diamino linker, and then pyreneacetic acid molecules are covalently bonded to the nanotube surface by a condensation reaction between the carboxylic groups and the surface amino groups. The fluorescent dye molecules were tracked in the samples using confocal laser scanning electron microscopy (CLSM) (λ_exc_ = 458 nm): CSLM images of the nanotubes synthesized with the P3CA-to-pyrrole molar ratios of (**a**) 1:15 and (**b**) 1:30. With permission from [19]; Copyright 2008, Wiley-VCH Verlag GmbH & Co. KGaA.

Various functional groups such as –COOH, –CN, –NH_2_, –SH can interact with metal cations through ion-ion or ion-dipole interactions. Conducting polymers containing either nitrogen or sulfur atoms in their repeating unit are attractive candidates for the preparation of metal/polymer hybrids. For example, polypyrrole (PPy) and polyaniline (PANI) have a nitrogen atom in their heterocyclic ring, and polythiophene (PTh) and poly(3,4-ethylenedioxythiophene) (PEDOT) have a sulfur atom in their heterocyclic ring. Thus, Ag, Au, and Pd nanoparticles could be readily deposited on the surface of PANI, PPy and PEDOT nanostructures without any dispersing or reducing agents [15–18]. On the other hand, monomers with a functional side group can be used to introduce desired functionality to the resulting polymers. Of course, it should be noted that the chemical/physical properties of monomers with a functional side group are different those of unfunctionalized, pristine monomers. Thus, it is important to judiciously choose detailed synthesis conditions for the successful polymerization. There are several good examples of using pyrrole derivatives to fabricate conducting polymer nanostructures with surface functional groups. First, pyrrole has been copolymerized with pyrrole-3-carboxylic acid (P3CA) in emulsion systems to fabricate tubular nanostructures [19–21]. The introduction of carboxylic groups was confirmed by immobilizing a fluorescent dye onto the nanotubes. As shown in Figure 1, the nanotubes exhibited cyan emission with uniform distribution over their surfaces, indicating that the carboxylic groups were thoroughly introduced into the polymer nanotubes.

**Figure 2.**
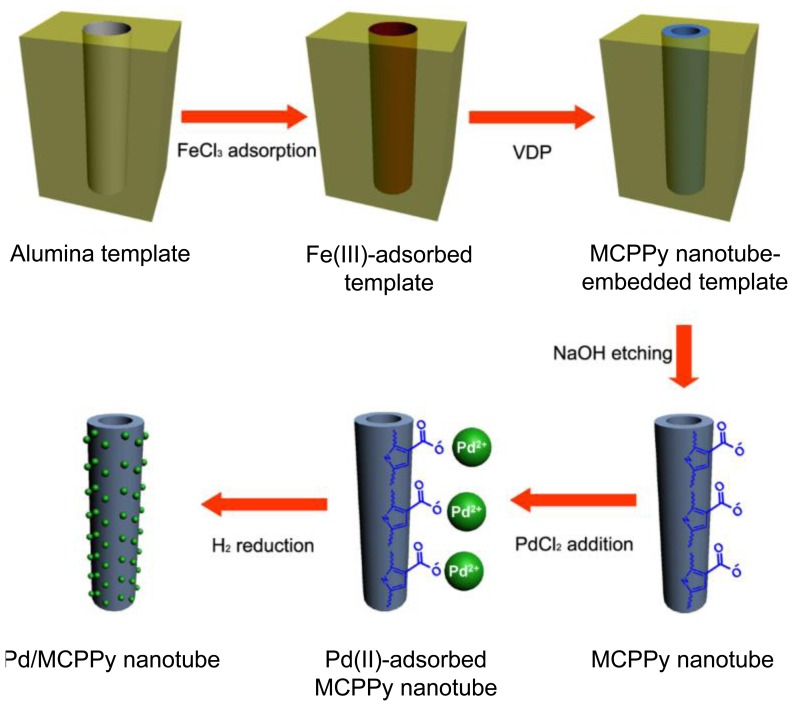
Schematic illustration of the fabrication of Pd nanoparticles-decorated MCPPy nanotubes. With permission from [22]; Copyright 2006, Wiley-VCH Verlag GmbH & Co. KGaA.

**Figure 3.**
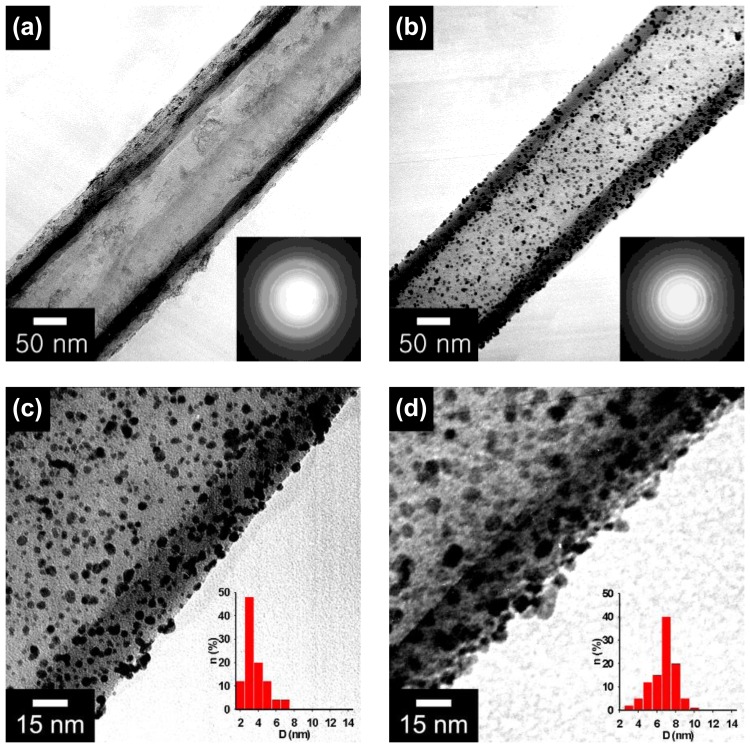
TEM images of (**a**) a MCPPy nanotube and (**b**) a Pd nanoparticles-decorated MCPPy nanotube prepared with a dilute PdCl_2_ solution. The inset images are the electron diffraction patterns; **(c**) and (**d**) magnified TEM images of Pd nanoparticles-decorated MCPPy nanotubes obtained with the palladium salt concentrations of 10^−4^ and 10^−3^ M, respectively. The Pd nanoparticle size is illustrated in the inset histogram. With permission from [22]; Copyright 2006, Wiley-VCH Verlag GmbH & Co. KGaA.

There is a notable example of fabricating metal nanoparticles-decorated PPy nanotubes using the above strategy [22]. Carboxylated pyrrole, P3CA monomers were chemically polymerized by ferric chloride within the cylindrical pores of a membrane template and magnetic phases were readily introduced into the nanotubes by precipitation of residual iron salt complexes during the template removal process. The main synthesis steps are represented in Figure 2. Magnetic carboxylated PPy (MCPPy) nanotubes were prepared by vapor-deposition polymerization (VDP) of P3CA monomers in porous aluminum template and subsequent chemical reduction of Fe(III) during the template etching process. The magnetic CPPy (MCPPy) nanotubes provide the anchoring sites for metal cations, thus making it possible to generate Pd nanoparticles on the nanotube surface at room temperature. Figure 3a shows transmission electron microscopy (TEM) image of MCPPy nanotubes with a diameter of *ca.* 100 nm and a wall thickness of 10–20 nm. The electron diffraction pattern indicated the presence of magnetites because only PPy is amorphous. Figure 3b–d exhibit TEM images of Pd nanoparticles-decorated MCPPy NTs prepared with different palladium salt (PdCl_2_) concentrations. As the palladium salt concentration increases, the diameter of the formed Pd nanoparticles turned out to increase from 3.6 up to 9.8 nm. It is considered that rapid nucleation relative to growth lead to a small particle size at low concentrations of Pd salt. Figure 3b–d demonstrate that relatively uniform Pd nanoparticles are formed on the surface of the MCPPy nanotubes. The Pd nanoparticles have a narrow size distribution (the inset histograms).

Silica nanoparticles were also attached to PPy nanotubes through a chemical binding between the amino group of silica nanoparticles and the carboxylic group of the nanotubes [23]. Furthermore, the carboxylic group can be modified to thiol group, which can be used as nucleation sites for the formation of metal or inorganic semiconductor nanoparticles. Typically, PbSe quantum dots could be decorated on the thiolated PPy nanotubes for photovoltaic application [24].

### (ii) Concurrent redox reactions

Most conducting polymers such as PPy, PANI, and PTh can be synthesized by oxidative polymerization using appropriate oxidizing agents. Normally, metal salts such as copper chloride, ferric chloride, ferric perchlorate, and ferric nitrate are widely using to obtain conducting polymers via chemical oxidation polymerization. Similarly, metal precursors containing iron, gold, silver, and platinum can be also used as oxidizing agents to achieve the oxidation polymerization of conducting polymer monomers, which allows the simultaneous formation of the metal and conducting polymer. Various nanoparticles have been synthesized using this approach, such as AgCl/PPy/chitosan nanospheres [25], gold/PANI hollow nanoparticles [26], gold/PEDOT nanoparticles [27], platinum/PEDOT nanoparticles [28], and silver/PPy nanostructures [29].

**Figure 4.**
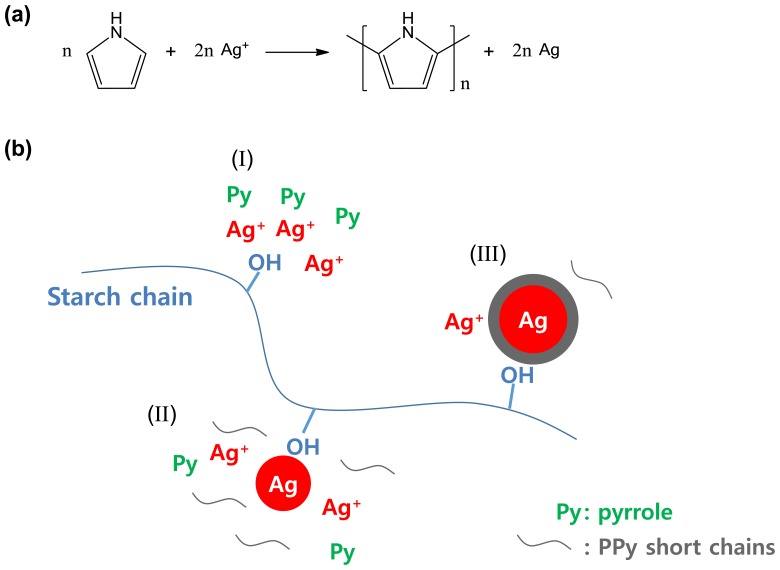
Schematic illustration of the formation mechanism of silver-PPy nanoparticles: (**a**) the scheme describing the chemical reaction between pyrrole and silver cation; (**b**) the reaction process could be divided into three stages (I, II, and III). Adapted with permission from [30]; Copyright 2012, American Chemical Society.

As a recent example, silver-PPy core-shell nanoparticles were readily obtained through the concurrent reduction/oxidation of silver nitrate/pyrrole in the presence of starch stabilizer [30]. The overall formation mechanism of the core-shell nanoparticles is described in Figure 4. First, pyrrole monomers are oxidized by silver cations, yielding PPy and silver atoms simultaneously (see Figure 4a). The polar groups of starch used as a steric stabilizer can act as nucleation sites to generate the core-shell nanoparticles at the initial stage (stage I). At the nucleation sites, silver cations are reduced to atomic silver by pyrrole monomers, whereas the pyrrole monomers are oxidized to pyrrole radical cations (stage II). Thus, the silver nanoseeds are being formed at the nucleation sites and coupling of pyrrole radical cations results in PPy short chains. Owing to the so-called common ion adsorption effect, additional silver cations are adsorbed onto the nanoseed surface that lead to the formation of a PPy shell through subsequent growth of surrounding PPy short chains (stage III). The resulting core-shell nanoparticles were *ca.* 36 nm in core diameter and *ca.* 13 nm in shell thickness, respectively. Importantly, those sequential reaction steps for yielding the core-shell nanoparticles are not complicated at all. The two reactants, pyrrole and silver nitrate are subjected to the successive reactions in a reactor to yield the final product, often referred to as one-pot synthesis.

The availability of this concurrent oxidation/reduction approach was also demonstrated in an interfacial polymerization system. Gold/PEDOT co-axial nanocables were fabricated without any special template by interfacial oxidative polymerization of 3,4-ethylenedioxythiophene (EDOT) in organic solution with a gold precursor, HAuCl_4_ in aqueous solution [31]. The nanocables had lengths of several micrometers, outer diameters *ca.* 50 nm, and central core *ca.* 30 nm. It is known that PEDOT has low band gap, high environmental stability, and high optical transparency in the oxidized, conductive state.

In the case of PANI, hydrogen peroxide was used as an oxidizing agent both to reduce a gold precursor (HAuCl_4_) to gold atoms and to polymerize aniline monomer, which is probably due to the relatively high oxidation potential of aniline monomer [32]. A new synthetic approach was used: namely, aniline is introduced in the vapor phase to an aqueous micelle solution where the reduction of the gold precursor is being performing by hydrogen peroxide. As a result, gold/PANI composite nanoparticles could be prepared, with the diameter of greater than 100 nm and the electrical conductivity of *ca.* 1.1 S·cm^−1^.

### (iii) Electrochemical deposition

**Figure 5.**
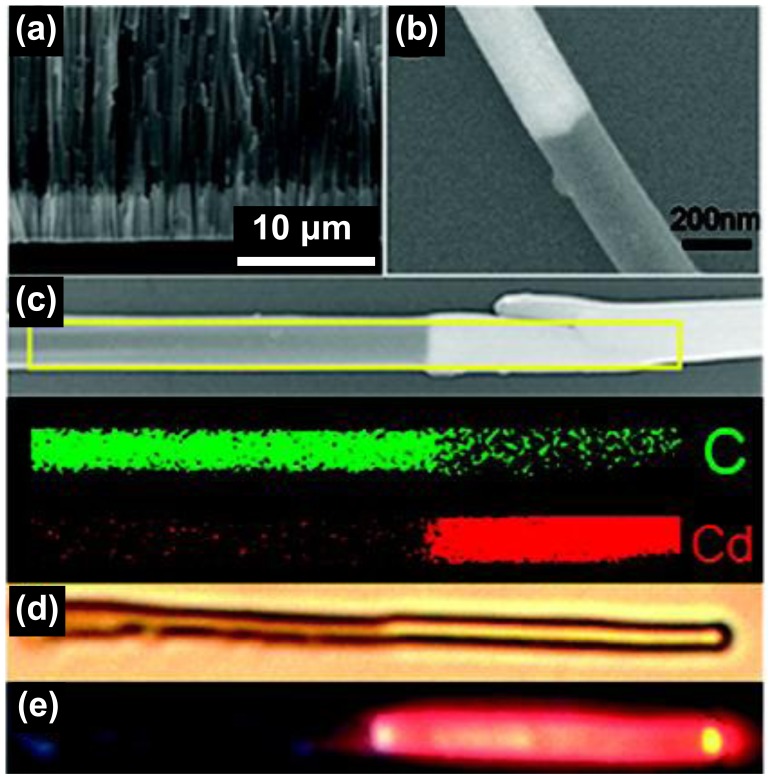
SEM images of CdS-PPy heterojunction nanowires: (**a**) side view image of CdS-PPy nanowires inside the alumina membrane template; (**b**) typical image of a single CdS-PPy nanowire; (**c**) element mapping of a single CdS-PPy nanowire; (**d**) optical image of a single CdS-PPy nanowire; (**e**) fluorescent image of a single CdS-PPy nanowire excited by a 405 nm laser. Adapted with permission from [33]; Copyright 2008, American Chemical Society.

In contrast with most polymers, the conductivity of conducting polymers makes them eligible for various electrochemical tool applications. As a typical example, conducting polymers can be readily obtained through electrochemical oxidation polymerization on electrodes. Electrochemical deposition involves a subsequent reduction of metal ions deposited from a solution onto an electrically conductive substrate. When conducting polymers are used as the conductive substrate, therefore, a variety of inorganic compounds can be introduced into the polymers using electrochemical deposition. Most related studies have used electrochemical co-deposition, for example, such as the electrochemical polymerization of a conducting polymer on an electrode, followed by the electrochemical deposition of an inorganic component. Li and co-workers reported the fabrication of CdS-PPy heterojunction nanowires by electrochemical co-deposition in porous alumina template (Figure 5) [33]. First, CdS was deposited into the cylindrical nanopores of the alumina membrane using CdCl_2_ and element sulfur as precursors in a dimethyl sulfoxide solution at a current density of 2.5 mA·cm^−1^. Subsequently, PPy was deposited in 0.1 M LiClO_4_ acetonitrile solution by applying a voltage of 0.85 V (*vs.* SCE). Scanning electron microscopy (SEM) images and element mapping demonstrated the formation of the heterojunction nanowires with diameters of 200–400 nm. Similarly, CdS-PANI nanowires and gold-PPy heterojunction nanorods have been also synthesized [34,35].

PPy/titania nanotubes were also obtained through sequential electrochemical processes [36]. Highly ordered titania nanotube array was first fabricated by an electro-oxidation of titanium sheet through an electrochemical anodization process and PPy was then deposited into the well-aligned titania nanotubes through a voltammetry deposition process, in which TiO_2_/Ti acted as a working electrode. The inner diameter, wall-thickness, and length of the titania nanotubes were 120–150 nm, 10–20 nm, and *ca.* 1 μm, respectively. The wall-thickness of the titania nanotubes was found to increase up to 50–80 nm after PPy deposition.

### (iv) Seeding approach

The so-called seeding approach has been widely used to produce micro- and nano-particles [37]. For example, magnetite (Fe_3_O_4_)/PEDOT core-shell nanoparticles could be synthesized through a seeding approach [38]. Magnetite nanoparticles were first prepared by a chemical precipitation method and were then used as nanoseeds for the polymerization of EDOT monomers. The magnetite nanoseeds were acid-etched, resulting in iron cations that can initiate the surface-confined oxidation polymerization. During this process, the polymerization kinetics affects highly the morphology of the final product at the nanometer regime. The polymerization rate of EDOT is relatively slow, which makes it possible to form ultrafine core-shell nanoparticles (*ca.* 13 nm in diameter) without interparticle aggregation.

**Figure 6.**
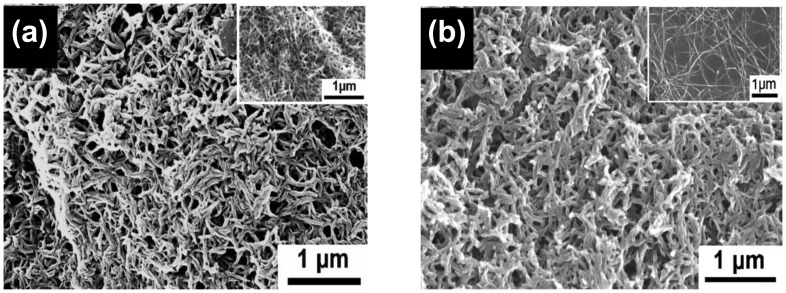
SEM images of PANI nanofibers synthesized with nanofiber seeds (insets): (**a**) single-walled carbon nanotubes; (**b**) V_2_O_5_ nanofibers. Adapted with permission from [39]; Copyright 2012, American Chemical Society.

One-dimensional nanostructures could be also obtained through seeding approaches. The Manohar and Zhang groups have also reported interesting results of fabricating PPy and PANI nanofibers using several different kinds of nanofiber seeds [39–41]. Nanofiber seeds such as V_2_O_5_ nanofibers and single-walled carbon nanotubes (SWNTs) acted as a reactive template that chemically interact with the monomer prior to the addition of initiator (Figure 6). Employing such nanofibers seeds has resulted in the formation of PPy or PANI composite nanofibers with *ca.* 20–50 nm, in which the morphology of the final product was dependent on the species, dimensions, and surface properties of seeds.

## 3. Conducting Polymer Nanohybrid-Based Sensors

### 3.1. Chemiresistive Gas Sensors

Various chemical species that can be harmful to human society or be used as diagnostic markers for disease, such as chemical weapons, toxic gases, and metabolites/volatile organic compounds (VOCs), have been found from the nature. For examples, nerve gas agents with odorless and colorless characteristics such as sarin, VX, soman, and tabun have are used as chemical weapons in the past wars. Organic compounds including ammonia, hydrogen chloride, and various hydroxyl acids are also characterized with colorless gas with a pungent and suffocating odor that is recognized as one of the primary irritants to human body. Moreover, metabolites compounds with extremely low concentrations (part-per-billions or part-per-trillions) in exhaled breath provide various disease informations or utilized for diagnostic markers. The level of ethane (*ca.* 800 pmol·L^−1^) and pentane in breath indicate high possibility in liver diseases and hepatic disease is marked by the presence of ammonia (*ca.* 23 μg %).

To detect or discriminate these chemical species, excellent technologies have been developed using various conducting polymer nanomaterials [10,11]. Multidimensional conducting polymer nanotubes were fabricated by electrospinning and vapor deposition polymerization processes, leading to the successful high-performance chemical nerve agent sensors [42]. Also, one-dimensional conducting polymer-based chemiresistive gas sensors were designed using microemulsion process that is one of the template-free methods, leading to the excellent chemiresistive transistor in sensor applications [43]. Although they displayed significant results in detectable sensing limitation, the advanced nanomaterials based on conducting polymers should be continuously developed for the current limitations such as low sensitivity and selectivity, response/recovery time, and complex pre-/post-process.

For the high-performance sensing devices, several important factors in sensing geometry design should be considered with as follows: (i) enlarged-surface areas, (ii) specific selectivity, and (iii) facile and portable geometry. From these fundamental conditions, inorganic/organic hybrid nanomaterials based on conducting polymers are one of the suitable transistors in chemiresistive gas sensor applications. It is caused that the conducting polymer nanohybrids can provide enhanced interaction and target selectivity by attaching or adsorbing pristine metal or metal-oxide nanomaterials.

#### 3.1.1. Nerve Agent Gas Detection

**Figure 7.**
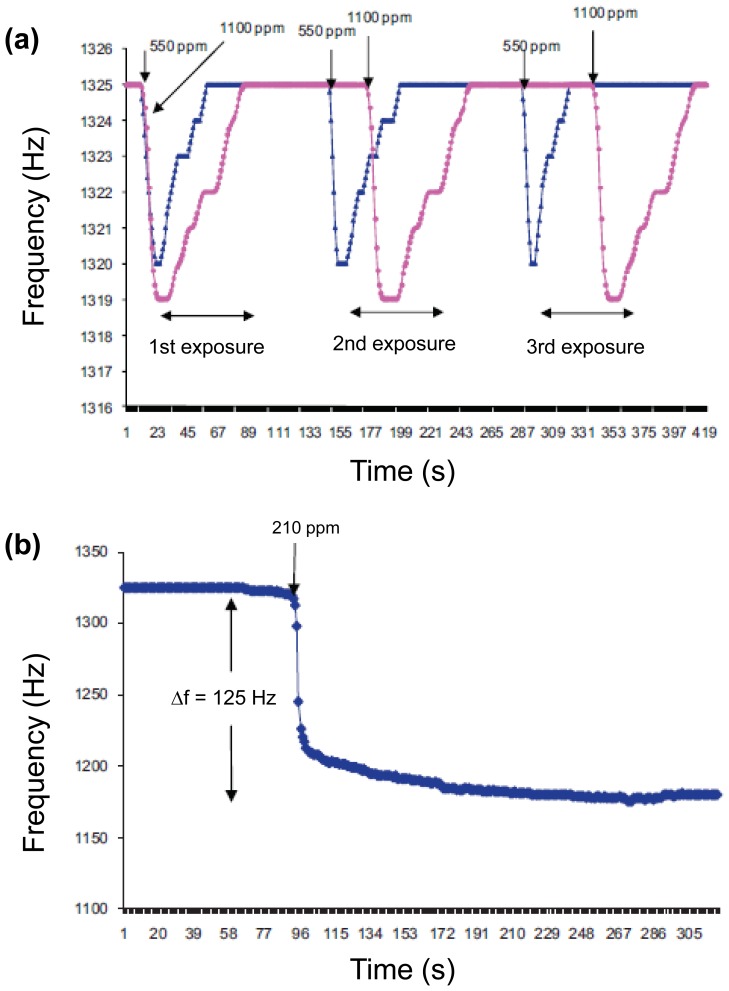
Responses of DMMP gas sensor toward (**a**) methanol and (**b**) DMMP. Adapted with permission from [45]; Copyright 2010, Elsevier B.V.

Nerve agents as organophosphate compounds induce the inhibition of serine protease in the nervous system and serious effects in human by reducing the breakdown of acetylcholine. There are various nerve agents. Especially, dimethyl methylphosphonate (DMMP), which is one of the diverse nerve gas simulants, has similar chemical structures with sarin gas that is used as a chemical weapon owing to its extreme potency as a nerve agent. Therefore, diverse technologies, including colorimetric assay, surface acoustic wave, and enzymatic assay, have been developed to detect DMMP gas. Representative researches toward nerve agent gas detection have been made with conducting polymers or carbon nanomaterials because of their excellent conductivity and facile functionalization, showing the high-performance sensing properties [42,44]. Especially, DMMP, which contains phosphoryl groups, can act as hydrogen-bond basicity, so that it easily can accept a proton from functionalized conducting polymer nanomaterials or nanohybirds. Recently, there was an interesting research using conducting polymer/metal hybrids without functional groups. Tiwari *et al.* fabricated PPy nanohydrids using electrochemical incorporation in presence of a cationic surfactant, cetyltrimethylammonium bromide (CTAB) [45]. Multidimensional PPy/copper phthalocyanine (CuPc)/CTAB/NaClO_4_ with unique flake-like CuPc substructure on the indium tin oxide substrate was demonstrated with infrared (IR) spectroscopy, cyclic voltammetry, and energy dispersive X-ray (EDX) analysis. Moreover, the flake-like CuPc can provide the enlarged surface area, leading to the higher sensitivity than that of flat structures. The sensing geometry using PPy nanohybrids with flake-like CuPc was developed in order to detect DMMP and toxic gases, which was connected to an acquisition system. This sensor showed significant frequency shift under various DMMP concentrations (Figure 7). Although the sensitivity from the PPy/copper nanohybrids was low compared to multidimensional conducting polymer nanostructures, they showed a possibility of detecting DMMP gases using metal nanohybrids.

#### 3.1.2. Toxic Gas Detection

To protect environment and human health from toxic gases, the sensing performance of toxic gas detectors has become increasingly important because low concentration of toxic gas can be even fatal. For example, hydrogen sulfide (H_2_S) from industrial processes is one of the highly toxic and corrosive gases and low concentrations (above 250 ppm) can lead to death [46]. Also, it can have potential use in terrorist attacks since it is colorless, flammable, and heavier than air [47]. Nitrogen oxides (NO, NO_2_, and N_2_O_4_) that produce the combustion of fuels can induce toxic acid rain, photochemical smog, and depletion of the ozone layer [48].

In the ammonia gas sensing, the most important issue is its fatal dose defined from Occupational Safety and Health Administration (U.S. Department of Labor, Washington, DC, USA) (15 min exposure limit for NH_3_ gas of 35 ppm by volume in the ambient air), leading to promoting the fabrication of advanced sensing geometries. From these concerns, various sensing materials which are inorganic or organic materials have been developed. Although they have attractive advantages including facile fabrication process, easy-controllable morphology, and low-cost, the limitations including high-temperature operation, slow response/recovery time, and low sensitivity remain as challenges.

**Figure 8.**
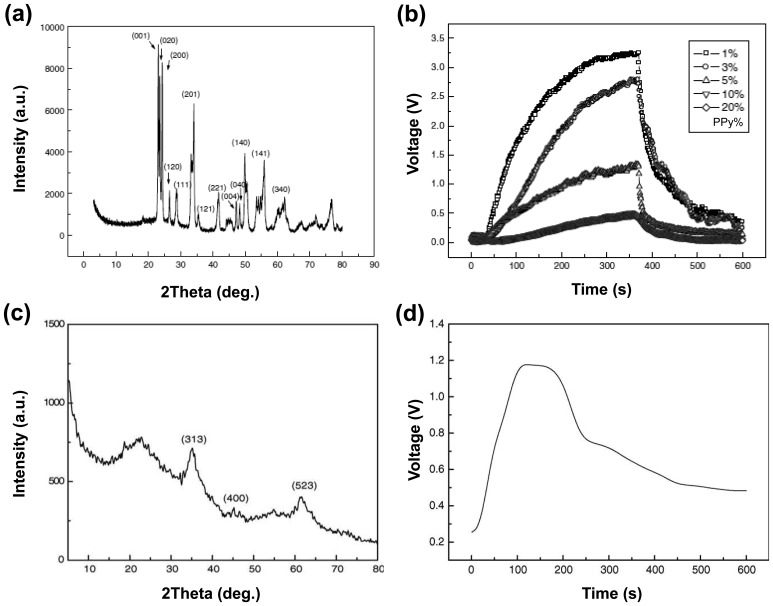
(**a**) XRD pattern of PPy/WO_3_ nanohybrids and (**b**) their response-recovery curves to H_2_S at 90 °C. Adapted with permission from Ref. [49]; Copyright 2006, Elsevier B.V. (**c**) XRD pattern (the three peaks correspond to γ-Fe_2_O_3_ phase) of PPy/γ-Fe_2_O_3_ nanohybrids annealed at 150 °C and (**d**) their response curve (voltage *vs.* time) to 0.1% H_2_S at 90 °C. Adapted with permission from [50]; Copyright 2006, Elsevier B.V.

Hydrogen sulfide can interact with PANI via a doping mechanism, which may result in a measurable change in PANI conductivity. However, H_2_S as a weak acid is partially doped into the polymer backbone, allowing only low sensitivity. To overcome this drawback, Virji *et al.* demonstrated the concept by incorporating transition-metal chlorides into PANI nanofibers and utilized as transistor for H_2_S detection (10 ppm) [47]. This is based on the formation of metal sulfide precipitates in metal salt solutions, giving rise to strong acids as the by-product. They provided experimental data to compare the sensing properties between pristine PANI and PANI/metal salts. Moreover, they prepared PANI nanofibers with various metal salts and characterized doping process using coordination of metal cations to the nitrogen atoms. The chemical sensors based on the PANI hybrids were measured the resistance changes upon the exposure to H_2_S (10 ppm). Also, they characterized the formation of sulfur in the films with EDX analysis. From the experimental results, the CuCl_2_ as metal source showed the strongest sensitivity compared to Zn and Cd metal salts. Compared to the effect of incorporated transition-metals, in sensing H_2_S using metal nanohybrids, another one of the important factors is a temperature because the activity of the metal substrates can be sensitive on temperature changes. Geng *et al.* also prepared PPy nanohybrids mixing mechanically with WO_3_ and showed excellent H_2_S detection at low temperature (90 °C) compared with conventional H_2_S sensors based on hybrids [49]. The PPy nanohybrids were characterized using HRTEM and X-ray diffraction (XRD) pattern, resulting that the average size of WO_3_ particles was *ca.* 29 nm calculated with Scherrer equation (Figure 8a). The voltage of the nanohybrid-based sensors was monitored by using a conventional circuit in which the sensor was connected with an external resistor in the testing circuit. In the sensing properties, the PPy nanohybrids showed stable reversibility and high sensitivity depending on the amount of PPy mass percent when operated at 90 °C. Moreover, they demonstrated that the mixing ratios of organic-inorganic hybrids can affect the sensing properties (Figure 8b). In addition, Geng *et al.* prepared PPy/γ-Fe_2_O_3_ nanohybrids using chemical oxidation with Fe(NO_3_)_3_·9H_2_O and used it for the detection of toxic gases [50]. The PPy-based nanohybrids were prepared by simultaneous gelling and polymerization under methoxy ethanol and Fe(NO_3_)_3_·9H_2_O. The nanohybrids were characterized by IR spectroscopy, thermogravimetric and differential thermal analysis, XRD analysis (Figure 8c), and electron microscopy. From these characterizations, the optimized annealing temperature and mole ratios of oxidation were selected and the operating temperature of the PPy/γ-Fe_2_O_3_ nanohybrids was studied at 90 °C. Using the PPy nanohybrids as transducer, they showed the sensitivity difference toward 0.1% H_2_S depending on the different mole ratios of hybrid composition and also demonstrated the maximized annealing temperature *vs.* H_2_S sensing properties in the PPy/γ-Fe_2_O_3_ nanohybrid system (Figure 8d). From these results, H_2_S sensors using conducting polymer/metals can be optimized with their kinds, detecting temperature, and annealing temperature.

**Figure 9.**
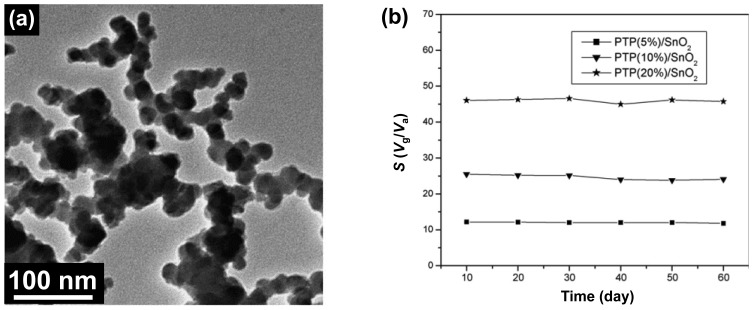
(**a**) TEM image of PTP (20%)/SnO_2_ composites; (**b**) The stability of PTP/SnO_2_ sensor to 100 ppm NO*_x_*. Adapted with permission from [54]; Copyright 2012, American Chemical Society.

Metal oxides such as SnO_2_, WO_3_, and ZnO have been utilized in monitoring NO_2_ gas [51–53]. However, they have low selectivity and sensitivity at ambient temperature. Therefore, organic-metal nanohybrids were developed to overcome their limitations. Kong *et al.* synthesized PTh/SnO_2_ nanocomposites by the *in situ* chemical oxidative polymerization and applied it for NO*_x_* detection [54]. The SnO_2_ particles were coated with PTh shell which was several thickness in nanoscale (Figure 9a). They found that the sensitivity and selectivity can increase by controlling PTh mass percent (1%, 5%, 10%, 20%, and 30%) in PTh/SnO_2_ nanocomposites. Based on the effects of p-n heterojunction between PTh and SnO_2_, the minimum detectable level (MDL) of nanocomposites toward NO*_x_* gas was *ca.* 10 ppm at room temperature, which was higher than pure SnO_2_. Moreover, the stability of PTh/SnO_2_ (PTh mass: 5%, 10%, and 20%) sensor was calculated during two months at room temperature, by which the enhanced stability was confirmed (Figure 9b). This NO*_x_* sensor using PTP/SnO_2_ nanocomposite operated high temperature (∼90 °C), while WO_3_-based transistors can detect NO*_x_* at low temperature. Using similar fabrication process, PTh/WO_3_ nanohybrids were also designed by Huang *et al.* and characterized with TEM, XRD analysis, and thermo-gravimetric analysis [55]. This nanohybrid showed higher thermal stability than pure PTh, leading to the utilization as sensing materials in chemical sensors. The MDL of the PTh/WO_3_ nanohybrids toward NO_2_ gas was a ppm level at low temperature. They demonstrated several critical factors, including the operating temperature and PTh contents, in conducting polymer/metal oxide-based chemical sensors. In this study, the 10 wt% PTh/WO_3_ nanohybrid showed the highest response at low operating temperature of 70 °C. Moreover, the NO*_x_* sensor at room temperature was demonstrated using PANI/In_2_O_3_ nanocomposites and they displayed high sensitivity. Sadek *et al.* created excellent NO_2_ gas sensor using PANI/In_2_O_3_ nanofiber composites [56]. The nanohybrids were fabricated by chemical oxidative polymerization of aniline in the presence of finely divided In_2_O_3_. The high-performance sensing geometry was developed by deposition of nanohybrids on surface acoustic wave (SAW), leading to the fast response and recovery times with good repeatability at room temperature. The conductivity of PANI/In_2_O_3_ increased with NO_2_ exposure, which indicates that NO_2_ gas can act as a dopant for the PANI/In_2_O_3_. Interestingly, based on this sensing mechanism, the MDL of PANI/In_2_O_3_ nanohybrid was *ca.* 510 ppb and rapid response/recovery time was calculated.

**Figure 10.**
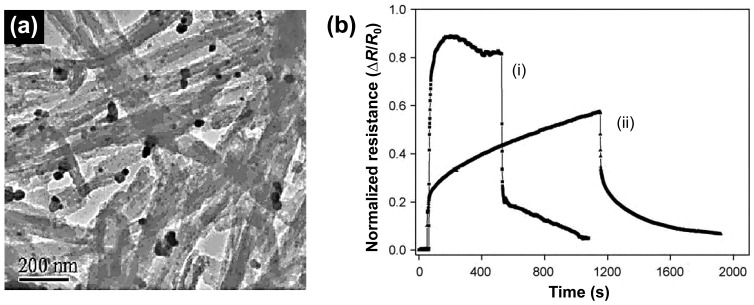

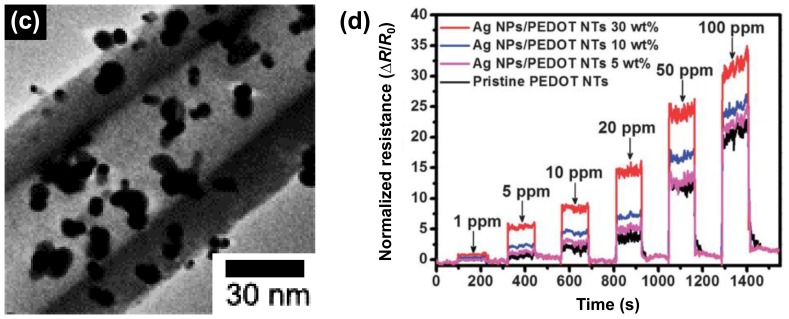
(**a**) TEM image of PPy/Ag-AgCl nanotubes; (**b**) Response of (i) PPy nanotubes and (ii) PPy/Ag-AgCl nanotubes upon exposure to 100 ppm of NH_3_. Adapted with permission from Ref. [58]; Copyright 2010, Elsevier B.V; (**c**) TEM images of silver/PEDOT nanotubes; (**d**) The sensing performances toward 1–100 ppm NH_3_ gases. Adapted with permission from [59]; Copyright 2012, Royal Society of Chemistry.

For the detection of NH_3_ gas, various materials and technologies have been explored. Among many different strategies, the combination of organic-inorganic nanomaterials displayed the most excellent sensing performances toward NH_3_ gas. Yang *et al.* provided a facile method in fabricating Ag/AgCl-decorated PPy (PPy/Ag-AgCl) nanotubes [57]. PPy nanotubes were fabricated through assembling process on the reactive self-degraded template with complex of ferric chloride and methyl orange. For uniform decoration of Ag and AgCl nanoclusters, PPy nanotubes were immersed in AgNO_3_ solution, which resulted in PPy nanotube hybrids, as shown in Figure 10a. The PPy nanotube hybrids were utilized as chemiresistors in chemical sensor applications and showed significant conductivity changes when NH_3_ gas (100 ppm) was exposed into the nanotube hybrids (Figure 10b). Moreover, the response time was *ca.* 150 s to reach the maximum and stable reproducible responses were also displayed. Several researchers also demonstrated that nanostructured metals deposited or chemically connected on the surface of the conducting polymers exhibits size-dependent sensing properties. Hong *et al.* reported the fabrication of a nanohybrids consisting of PPy and Pd through thermal dynamic refluxing of Pd salt and subsequent encapsulating of formed Pd nanoparticles with a PPy shell via vapor deposition polymerization [58]. The nanohybrids designed with synthetic variables, including reaction time and polymer additives, were characterized by TEM and atomic force microscopy, and then they were utilized as the chemiresistor in NH_3_ sensing system. The electrical responses from Pd-encapsulated PPy nanoparticles toward NH_3_ were highly fast and stable at the range of the 50–200 ppm NH_3_ gases. Moreover, the response and recovery time were 14 s and 148 s for 1000 ppm NH_3_, respectively. Judging from these results, the size of Pd nanoparticles and the morphology of the hybrid films are important to achieve high-performance ammonia sensors based on the nanohybrids. As advanced nanohybrids for NH_3_ sensing materials, Park *et al.* constructed silver nanoparticles-decorated PEDOT nanotubes using a modified concurrent redox reaction in porous alumina membrane [59]. To fabricate the PEDOT nanotube hybrids, the alumina template was immersed into Fe(NO_3_)_3_ and AgNO_3_ solutions and vaporized EDOT monomer was introduced into the template membrane under 10^−2^ Torr at 60 °C for 3 h. Finally, PEDOT nanohybrids were successfully obtained after etching the template membrane. To characterize these nanohybrids, various analysts including TEM, SEM, and XRD analysis were employed (Figure 10c). The diameter and wall-thickness of PEDOT nanohybrids were *ca.* 100 and *ca.* 25 nm, and the size of the decorated silver nanoparticles was *ca.* 10 nm. The PEDOT nanotube hybrids were utilized as the chemiresistor in ammonia sensor by virtue of the enhanced conductivity and high affinity toward NH_3_ by silver nanoparticles. The highest sensitivity and rapid response time toward NH_3_ (1 ppm and less than 1s) can be obtained from PEDOT nanohybrids owing to the silver nanoparticles dispersed on the surface of PEDOT nanotubes. Moreover, the optimal deposited amount of silver nanoparticles were also determined by adjusting AgNO_3_ concentration (Figure 10d). The sensing geometry was constructed by the deposition of nanohybrids on the electrodes and induced the resistance changes when NH_3_ was adsorbed on the surface of nanohybrids. They showed comprehensive sensing properties using experimental date: (i) the sensitivity and selectivity changes dependence upon amount of Ag NPs on the surface of PEDOT nanotubes, and (ii) response and recovery time upon increasing NH_3_ concentrations.

**Figure 11.**
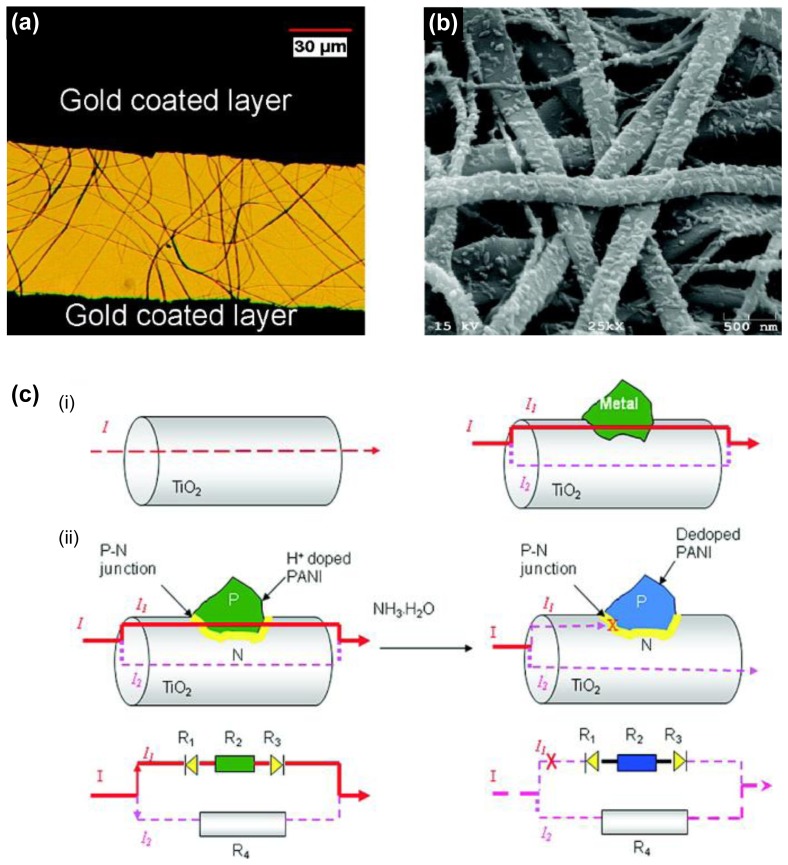
(**a**) An optical microscope image of the sensor and (**b**) SEM image of titania/PANI nanofiber hybrids. (**c**) Scheme of nanosized p-n heterojunction as a switch to control the electric current flow in titania nanofibers. Adapted with permission from [60]; Copyright 2010, American Chemical Society.

In the detection of ammonia gas, the p-n junctional type as transistor was introduced for the first time. Gong *et al.* fabricated unique conducting polymer nanograins using PANI and electrospun titania fibers, leading to the ultrasensitive NH_3_ gas sensor (Figures 11a,b) [60]. They introduced p-n heterojunctions combining p-type PANI and an electrospun n-type semiconductive titania nanofiber using immersing process. Moreover, the chemiresistive ammonia sensor based on titania/PANI nanohybrids was developed with electric current switches that can be operated by the resistance change from p-n heterojunction when NH_3_ gas is absorbed by PANI nanograins (Figure 11c). Surprisingly, the MDL of chemiresistive sensor was *ca.* 50 ppt NH_3_ gas in air, which is 1000 times more sensitive than the best PANI sensor reported in the literature.

#### 3.1.3. Volatile Organic Compound Detection

**Figure 12.**
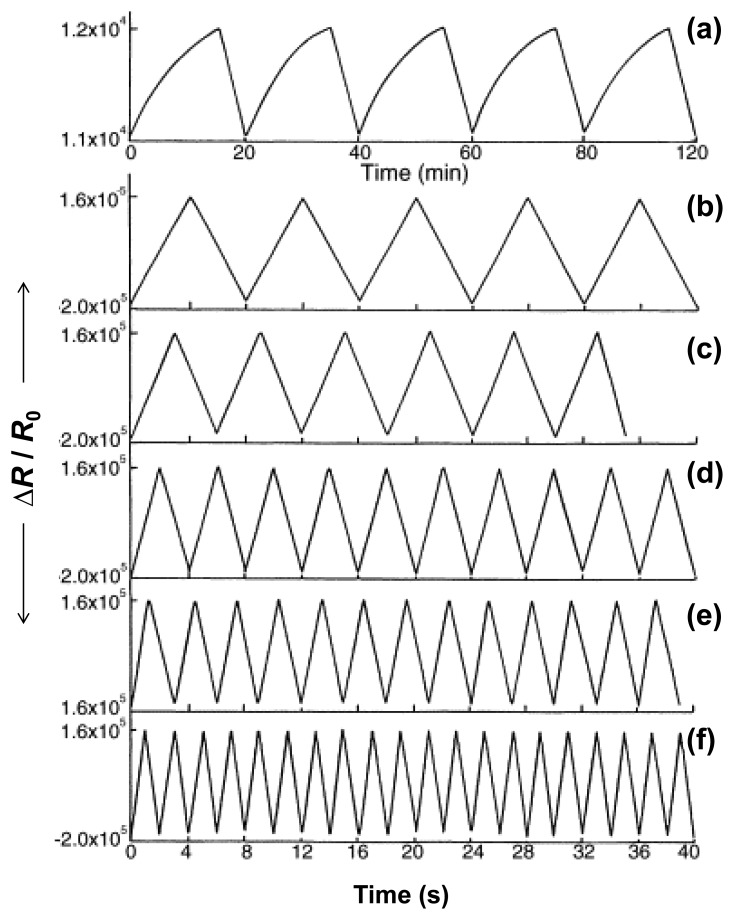
(**a**) Response curves of (**a**) PANI_only and (**b**–**f**) Pd-PANI nanohybrids toward different methanol concentrations: (**a**) 2000 ppm; (**b**) 1 ppm; (**c**) 5 ppm; (**d**) 10 ppm; (**e**) 100 ppm; and (**f**) 2000 ppm. Adapted with permission from [63]; Copyright 2006, Elsevier B.V.

There are a variety of VOCs, including alkanes and benzene derivatives, to which we are exposed our daily life. Moreover, extremely low concentrations (ppb to ppt) of metabolites or VOCs can be used as diagnostic markers for diseases [61]. Contents over certain amount of ethanol (*ca.* 800 pmol·L^−1^) can be diagnosed as liver diseases and, in case of lung cancer, 22 VOCs can be found in the breath from patients [62]. Therefore, various detection tools, such as gas/liquid chromatography, mass spectroscopy, and chemical treatments, have been explored to discriminate exhaled breath. Although the detection tools have individual advantages, their limitations in terms of sensitivity and selectivity still remained as challenges because the chemical structures of VOCs are almost similar or slightly have atomic-differences. Recently, many researchers have tried to overcome the limitations using conducting polymer nanohybrids owing to their specificity toward alkanes. Athawale *et al.* successfully obtained Pd-PANI nanohybrids using oxidation polymerization with aniline solution containing Pd nanoparticles [63]. The nanohybrids can induce specific interaction between the methanol molecules with the imine nitrogen. Specifically, the positive charge on the imine nitrogen is reduced by interacting with methanol in the presence of Pd nanoparticles, resulting in that the imine nitrogen was converted to amine. Based on this sensing mechanism, the Pd-PANI nanohybrids had highly selective and sensitive to methanol vapors (Figure 12). Interestingly, their specific selectivity was also demonstrated by exposing to mixtures including methanol-ethanol and methanol-isopropanol. Moreover, the methanol sensors based on the nanohybrids displayed a stable response for a long time.

Choudhury also demonstrated highly specific ethanol sensors using silver/PANI nanohybrids that were prepared by *in situ* oxidative polymerization of aniline monomer in the presence of different contents of silver nanoparticles [64]. The formation of the nanohybrids can be easily controlled by adjusting silver concentrations owing to the aggregation effect. They showed comparison analysis between pure PANI and silver/PANI nanohybrids. The most outstanding feature was their conductivity difference, which affects the sensitivity toward ethanol sensitivity. The PANI nanohybrids had superior ethanol sensing capacity compare to pure PANI. Moreover, the sensitivity showed linear relationship between ethanol and silver concentrations. The PANI nanohybrid sensors also provided reproducible sensing properties.

### 3.2. Biosensors

One of the great goals in developing the biological sensing materials is to fabricate rapid, accurate, and portable sensing geometry or diagnostic technologies for the prevention of fatal disease. Various sensing nanomaterials such as graphene, carbon nanotubes, metal transistors, and nanohybrids have been developed. Especially, conducting polymer-based nanohybrids have been studied to detect directly or indirectly related-biomolecules, such as hydrogen peroxide, glucose, dopamine, DNA, and proteins, by virtue of their electrical conductivity, affinity toward biomolecules, and biocompatibility.

#### 3.2.1. Glucose Detection

Incorporation of biomolecules as the recognition element into signal transducers can enable to detect the target molecules. A variety of biosensors based on transducers conjugated with bioreceptors such as antibodies, aptamers, cells, and enzymes have been developed. In particular, for diagnosing diabetes, amperometric glucose biosensors have been constructed with glucose oxidase (GOx) electrodes that can induce enzymatic regeneration reaction, leading to quantitative information on the glucose level. First, the controlled loading amount of GOx can affect the sensing properties of glucose sensors. Xian *et al.* reported a novel glucose biosensor based on gold nanoparticles-conjugated PANI nanofiber hybrids immobilized with GOx [65]. The PANI nanofiber hybrids provided fascinating advantages, including enlarged surface areas, excellent conductivity, and many micron-sized gaps to easily immobilize GOx. Moreover, this enzyme-conjugated nanohybrids allow the rapid transmit electron and enhanced interactions toward various glucose concentrations. To characterize their sensing operation, first of all, several experiments were performed to determine the electrochemical response of H_2_O_2_ at the PANI nanofiber hybrids. PANI nanofiber hybrids displayed the most efficient sensing properties toward H_2_O_2_ because incorporation of gold nanoparticles in PANI nanofibers enabled the electron transfer between electrodes and H_2_O_2_. To optimize sensing operation, several experimental factors (pH and loading amount of GOx) were also characterized. Based on these results, the sensing properties of PANI nanohybrids were further examined to evaluate glucose sensor performance. In real samples, this glucose sensor showed rapid response time (less than 5 s) and significant current changes toward various glucose concentrations. Moreover, excellent selectivity from the real-sample was also displayed. Second, to obtain enhanced sensitivity, the measurement conditions are considered. Xu *et al.* also fabricated enzyme-immobilized conducting polymer nanohybrids to construct glucose biosensor [66]. They fabricated multiwalled-carbon nanotubes (MWNTs) coated with PANI and dendrimer-encapsulated Pt nanoparticles (Pt-DENs) by in site polymerization and immobilized GOx on the nanotube surface. The glucose biosensors using PANI nanohybrids was examined with important factors (pH and potentials), by which the optimized operating condition was determined from stable responses. Moreover, the attached Pt-DENs can accelerate electron transfer and aid the immobilization of enzyme, helping their sensing performances. To confirm the biosensing performance using PANI nanohybrids, the real-time responses of the nanohybrids-based electrode were demonstrated with the addition of various glucose concentrations. The significant current changes were displayed within 5 s. Its MDL was *ca.* 0.5 μM and saturated over 16 mM. Moreover, the influence of common interfering electroactive substances such as uric acid, acetaminophen, and ascorbic acid was also characterized for excellent selectivity. The PANI nanohybrids have shown excellent electrochemical properties owing to their electrochemical doping and re-doping behavior under acid and base condition. Especially, the stable electrical properties of the nanohybrids make them suitable for biosensing. Liu *et al.* reported the formation of novel PANI-coated Fe_3_O_4_ nanoparticles-MWNT nanohybrids by the co-precipitation of Fe(III) and Fe(II), followed by in site polymerization [67]. The PANI nanohybrids were characterized by IR spectroscopy, electron microscopy, XRD analysis and thermogravimetric analysis, which proved their successful fabrication. Also, the magnetic properties of PANI nanohybrids were confirmed by hysteresis loops of Fe_3_O_4_ nanoparticles and PANI-Fe_3_O_4_-MWNT nanohybrids. These nanohybrids were utilized for glucose biosensor by electrochemical doping of GOx. The glucose biosensor showed rapid dynamic equilibrium within 5–6 s and stable reproducibility toward 1 mM glucose.

Lastly, the metal density also becomes a one of important factors in glucose sensors. Guo *et al.* also reported a simple and facile procedure for fabricating PANI nanohybrids for high-efficiency electrocatalysts [68]. They prepared PANI nanofiber-supported high-density Pt nanoparticles at room temperature, which had a couple of benefits such as high-density of Pt nanoparticles without linkers, free-template at room temperature, and high-performance electrochemical devices. The PANI nanohybrids, which were fabricated by a simple wet-chemical approach, were combined with GOx to utilize as nanoprobes of glucose biosensor. The PANI nanohybrids-based glucose biosensor showed highly sensitive response toward various glucose concentrations owing to enhanced electron transfer. Recently, Bian *et al.* provided enzyme-free electrochemical biosensing toward hydrogen peroxide. This methodology provide cost-effective and easy-fabrication process. They fabricated Pt/PPy hollow hybrids using wet-chemical method via magnetite template and applied them into the H_2_O_2_ biosensors [69]. The cyclic measurements from Pt/PPy hollow nanosphere hybrids exhibited good electrocatalytic activity towards the reduction of H_2_O_2_ in a buffer solution (pH = 7.0). The response from hybrid-based biosensor was less than 2 s with linear range of 1.0–9.0 mM and the MDL was 1.2 μM (signal to noise: 3).

#### 3.2.2. Dopamine Detection

**Figure 13.**
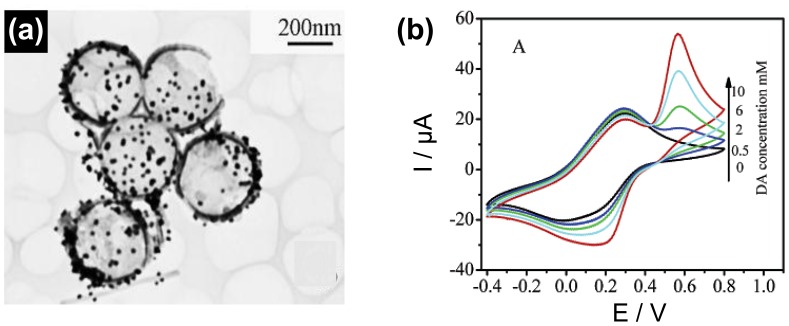
(**a**) TEM image and (**b**) cyclic voltammograms of gold/PANI hollow nanosphere nanohybrids toward various dopamine concentrations. Adapted with permission from [26]; Copyright 2006, American Chemical Society.

Dopamine that belongs to excitatory chemical neurotransmitters is one of the most significant catecholoamines and affects to the central nervous and hormone systems. Abnormal dopamine concentration in human body can be used as diagnosis measurement of fatal diseases such as Parkinson's and Alzheimer's diseases. Therefore, efficient sensing methodologies have been designed to detect dopamine molecules. Feng *et al.* fabricated gold/PANI hollow nanosphere hybrids using polystyrene-sulfonated polystyrene core-shell gel particle templates [26]. Moreover, they controlled the shell-thickness and amount of gold nanoparticles decorated on the surface of PANI shell, leading to the advanced transistor in the range of the sensor applications (Figure 13a). Especially, the conductivity of the PANI nanohybrids was more than 3 times higher than that of the conventional PANI hollow sphere. Based on excellent electrical properties, the nanohybrids were applied for dopamine detection. Also, gold/PANI hybrid system can provide redox behavior in different pH buffer solutions because gold nanoparticles facilitate the electrocatalytic oxidation of dopamine, resulting to efficient dopamine biosensor. The MDL of gold/PANI nanohybrids was *ca.* 2 mM dopamine concentration, which is *ca.* three times higher than that in the PANI system (Figure 13b). Compared to conventional dopamine biosensors, Li *et al.* proposed a novel sensing platform based on gold nanoparticles/PANI/polydopamine hybrids [70]. The nanohybrids were synthesized by facile processes: (i) spontaneous self-polymerization of dopamine, (ii) the formation of gold nanoparticles with both polydopamine and aniline by reducing agent, leading to the excellent electroactivity in neural solution. Importantly, the role of the gold nanoparticles in the nanohybrids is to enhance catalytic ability. Under optimized conditions, the nanohybrid sensor showed rapid response (4 s) and ultrasensitive DML (4.0 × 10^−7^ M) to ascorbic acid. Recently, graphene-based dopamine sensor was introduced and displayed high sensitivity owing to its high conductivity and enlarged surface areas. Ultrasensitive dopamine detector was created by Wu and Shen [71]. They fabricated gold nanoparticles decorated PPy/graphene oxide nanohybrids using in-situ chemical oxidative polymerization. The gold@PPy/graphene oxide nanohybrids were characterized by electron microscopy and X-ray photoelectron spectroscopy (XPS). The nanohybrids displayed a crumpled and wrinkled surface, which can provide enhanced surface areas, and have uniform flower-like gold nanoparticles, leading to the ultrasensitive MDL (18.29 pM) with a linear range of 1–5000 nM for dopamine.

#### 3.2.3. DNA Detection

**Figure 14.**
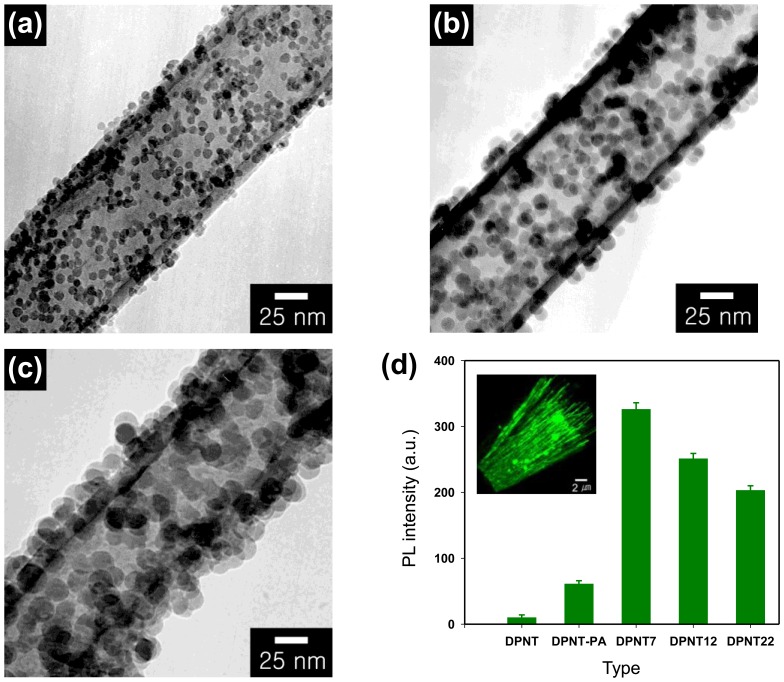
TEM images of PPy nanotubes with different sized amine-modified silica nanoparticles attached: (**a**) 7 nm; (**b**) 12 nm; and (**c**) 22 nm; (**d**) photoluminescence intensity of pyreneacetic acid conjugated with amine groups on the nanotube hybrids (DPNT: unconjugated PPy nanotube hybrids; DPNT-PA: the nanotube hybrid incubated with pyreneacetic acid in the absence of a condensing agent; DPNT7: the nanotube hybrids formed using 7 nm silica; DPNT22: the nanotube hybrids formed using 22 nm silica (inset: CLSM image of pyreneacetic acid conjugated with amine groups of the nanotube hybrids. Adapted with permission from [23]; Copyright 2006, Wiley-VCH Verlag GmbH & Co. KGaA.

Nucleic acids such as DNA or RNA can affect important protein information in human body. As a results, various DNA analysis have been reported with advanced sensing devices in related applications including infectious diseases, drug discoveries, and clinical diagnosis. One of the attractive DNA sensing systems is electrochemical detectors based on biomaterial-conjugated conductive geometries. Electrochemical DNA sensors showed direct and fast electrical-signals toward target biomolecules, leading to the simple sensing geometries without complex signal transduction equipment and expensive analyzers. Moreover, the hybridized transducers in DNA sensors showed more sensitive and selective sensing performances compared to pristine them. Especially, DNA detectors based on conducting polymer nanohybrids provided excellent electrical properties, resulting that high-performance DNA sensors were constructed. Tian *et al.* reported stable multilayer films using PANI/mercaptosuccinic-acid-capped gold (MSAG) nanoparticles hybrids by layer-by-layer method [72]. PANI can provide the excellent redox activity by molecular doping and shift their neutral conditions to significant redox position, leading to the excellent electrochemical sensing devices. In this study, the PANI layer was easily stimulated by modified MSAG under no polyelectrolytes conditions and its electroactivity was shifted to neutral pH without polyelectrolytes. Moreover, PANI hybrid layer was functionalized to introduce the amino-terminated DNA catcher probes (NH_2_-DNA), which can be used for monitoring their hybridization with different DNA target strands under electrochemical methods. Based on the electrochemical impedance spectroscopy (EIS), the surface-charge density of the PANI hybrids increased from the hybridization process with target DNA, resulting that the electrostatic repulsion also increased. Therefore, the comparison between before and after the introduction of target DNA (500 nM) can be monitored with EIS spectra. Jang *et al.* reported excellent dual-functionalized conducting polymer nanohybrids substrates for molecular-probe and DNA-carrier [23]. They, for the first time, constructed bifunctionalized conducting polymer nanotube hybrids using VDP in ferric chloride-adsorbed alumina membranes with 100 nm pore diameter. Moreover, the conducting polymer nanotubes were also functionalized with carboxylic groups by the polymerization of pyrrole-2-carboxylic acid. Functionalized PPy nanotubes can be easily decorated a photoluminescent molecule, pyreneacetic acid, which silica nanoparticles were employed as the linker. The silica nanoparticles with surface amino groups were first attached to the PPy nanotubes and then pyreneacetic acid was linked with the silica nanoparticles. Furthermore, single-stranded DNA (ssDNA) with a terminal amino group was attached to the residual carboxyl groups of the nanotubes. The resulting PPy nanotube hybrids were characterized with IR spectroscopy, XPS, and electron microscopy. Interestingly, the size of the attached silica-NH_2_ nanoparticles decreased with increasing number of the nanoparticles, resulting in that the total surface area of silica nanoparticles increased (Figure 14a–c). The PPy nanotubes hybrids with silica nanoparticles were found to have more sensitive responses than only PPy nanotubes owing to the enhanced surface area. The nanotube hybrids showed excellent PL intensity (Figure 14d) and different intensities depending on the size of the attached silica nanoparticles (7 nm > 12 nm > 11 nm). Feng *et al.* also fabricated DNA sensors using gold/PANI nanotubes hybridized membranes. The PANI nanohybrids were constructed on the glass carbon electrode and utilized as electrochemical sensing geometry to detect the immobilization and hybridization of DNA. The cyclic voltammetry, pulse voltammetry, and electrochemical impedance spectroscopy were introduced to characterize PANI nanohybrids. Based on the DNA probes using PANI nanohybrids, they showed excellent recognition of DNA hybridization under the phosphinothricin acetyltransferase gene (PAT). The DML toward the sequence-specific DNA was 3.1 × 10^−13^ mol·L^−1^, which is high-performance DNA sensor compared to conventional DNA biosensors. Moreover, specific recognition and reproducibility of the DNA sensor using PANI nanohybrids were also evaluated.

## 4. Conclusions and Outlook

The purpose of this review article is to provide a better understanding for the recent research trend of conducting polymer-based nanohybrid sensors. Conducting polymer-based nanohybrids have the strong potential to provide distinct properties that were not found in the individual component and their bulk counterparts. However, on the other hand, it seems like that there has been relatively limited research on combining of conducting polymers with inorganic materials on nanoscale, except carbon nanomaterials, which is presumably due to the difficulties in obtaining well-defined polymer nanostructures. Although there have been successful trials that are highlighted in here, the absence of various material candidates is considered to retard the wide application of the conducting polymer nanohybrids to sensors. There is no doubt that new or improved chemical and physical properties from the conducting polymer nanohybrids can be exploited for fabricating high-performance sensors. Accordingly, we believe that further advance in synthesis techniques of conducting polymer nanohybrids will lead to a huge leap of sensor technology.

## References

[1] Janaky C., Visy C. (2013). Conducting polymer-based hybrid assemblies for electrochemical sensing: A materials science perspective. Anal. Bioanal. Chem..

[2] Hangarter C.M., Chartuprayoon N., Hernandez S.C., Choa Y., Myung N.V. (2013). Hybridized conducting polymer chemiresistive nano-sensors. Nano Today.

[3] Liu Z., Zhang L., Poyraz S., Zhang X.Y. (2013). Conducting polymer-metal nanocomposites synthesis and their sensory applications. Curr. Org. Chem..

[4] Yoon H.S., Choi M.J., Lee K.A., Jang J.S. (2008). Versatile strategies for fabricating polymer nanomaterials with controlled size and morphology. Macromol. Res..

[5] Jang J. (2006). Conducting polymer nanomaterials and their applications. Adv. Polym. Sci..

[6] Gangopadhyay R., De A. (2000). Conducting polymer nanocomposites: A brief overview. Chem. Mater..

[7] Oh W.K., Kwon O.S., Jang J. (2013). Conducting polymer nanomaterials for biomedical applications: Cellular interfacing and biosensing. Polym. Rev..

[8] Park H.W., Kim T., Huh J., Kang M., Lee J.E., Yoon H. (2012). Anisotropic growth control of polyaniline nanostructures and their morphology-dependent electrochemical characteristics. ACS Nano.

[9] Yoon H., Hong J.Y., Jang J. (2007). Charge-transport behavior in shape-controlled poly (3,4-ethylenedioxythiophene) nanomaterials: Intrinsic and extrinsic factors. Small.

[10] Yoon H., Jang J. (2009). Conducting-polymer nanomaterials for high-performance sensor applications: Issues and challenges. Adv. Funct. Mater..

[11] Yoon H. (2013). Current trends in sensors based on conducting polymer nanomaterials. Nanomaterials.

[12] Lu X.F., Zhang W.J., Wang C., Wen T.C., Wei Y. (2011). One-dimensional conducting polymer nanocomposites: Synthesis, properties and applications. Prog. Polym. Sci..

[13] Rajesh, Ahuja T., Kumar D. (2009). Recent progress in the development of nano-structured conducting polymers/nanocomposites for sensor applications. Sens. Actuator B Chem..

[14] Park S.J., Kwon O.S., Lee S.H., Song H.S., Park T.H., Jang J. (2012). Ultrasensitive flexible graphene based Field-Effect Transistor (FET)-type bioelectronic nose. Nano Lett..

[15] Tseng R.J., Huang J.X., Ouyang J., Kaner R.B., Yang Y. (2005). Polyaniline nanofiber/gold nanoparticle nonvolatile memory. Nano Lett..

[16] Zhang X.Y., Manohar S.K. (2005). Narrow pore-diameter polypyrrole nanotubes. J. Am. Chem. Soc..

[17] Zhang X.Y., Lee J.S., Lee G.S., Cha D.K., Kim M.J., Yang D.J., Manohar S.K. (2006). Chemical synthesis of PEDOT nanotubes. Macromolecules.

[18] Qin X.Y., Lu W.B., Luo Y.L., Chang G.H., Sun X.P. (2011). Preparation of Ag nanoparticle-decorated polypyrrole colloids and their application for H_2_O_2_ detection. Electrochem. Commun..

[19] Yoon H., Kim J.H., Lee N., Kim B.G., Jang J. (2008). A novel sensor platform based on aptamer-conjugated polypyrrole nanotubes for label-free electrochemical protein detection. ChemBioChem.

[20] Yoon H., Lee S.H., Kwon O.S., Song H.S., Oh E.H., Park T.H., Jang J. (2009). Polypyrrole nanotubes conjugated with human olfactory receptors: High-performance transducers for FET-type bioelectronic noses. Angew. Chem. Int. Ed..

[21] Yoon H., Jang J. (2008). A field-effect-transistor sensor based on polypyrrole nanotubes coupled with heparin for thrombin detection. Mol. Cryst. Liq. Cryst..

[22] Ko S., Jang J. (2006). A highly efficient palladium nanocatalyst anchored on a magnetically functionalized polymer-nanotube support. Angew. Chem. Int. Ed..

[23] Jang J., Ko S., Kim Y. (2006). Dual-functionalized polymer nanotubes as substrates for molecular-probe and DNA-carrier applications. Adv. Funct. Mater..

[24] Kim J.S., Kim W.J., Cho N., Shukla S., Yoon H., Jang J., Prasad P.N., Kim T.D., Lee K.S. (2009). Synthesis and properties of quantum dot-polypyrrole nanotube composites for photovoltaic application. J. Nanosci. Nanotechnol..

[25] Cheng D.M., Xia H.B., Chan H.S.O. (2004). Facile fabrication of AgCl@polypyrrole-chitosan core-shell nanoparticles and polymeric hollow nanospheres. Langmuir.

[26] Feng X.M., Mao C.J., Yang G., Hou W.H., Zhu J.J. (2006). Polyaniline/Au composite hollow spheres: Synthesis, characterization, and application to the detection of dopamine. Langmuir.

[27] Li X.H., Li Y.C., Tan Y.W., Yang C.H., Li Y.F. (2004). Self-assembly of gold nanoparticles prepared with 3,4-ethylenedioxythiophene as reductant. J. Phys. Chem. B.

[28] Liu Y., Lu N., Poyraz S., Wang X.L., Yu Y.J., Scott J., Smith J., Kim M.J., Zhang X.Y. (2013). One-pot formation of multifunctional Pt-conducting polymer intercalated nanostructures. Nanoscale.

[29] Wang S.B., Shi G.Q. (2007). Uniform silver/polypyrrole core-shell nanoparticles synthesized by hydrothermal reaction. Mater. Chem. Phys..

[30] Chang M., Kim T., Park H.W., Kang M., Reichmanis E., Yoon H. (2012). Imparting chemical stability in nanoparticulate silver via a conjugated polymer casing approach. ACS Appl. Mater. Inter..

[31] Lu G.W., Li C., Shen J.Y., Chen Z.J., Shi G.Q. (2007). Preparation of highly conductive gold-poly(3,4-ethylenedioxythiophene) nanocables and their conversion to poly (3,4-ethylenedioxythiophene) nanotubes. J. Phys. Chem. C.

[32] Sarma T.K., Chattopadhyay A. (2004). One pot synthesis of nanoparticles of aqueous colloidal polyaniline and its Au-nanoparticle composite from monomer vapor. J. Phys. Chem. A.

[33] Guo Y.B., Tang Q.X., Liu H.B., Zhang Y.J., Li Y.L., Hu W.P., Wang S., Zhu D.B. (2008). Light-controlled organic/inorganic P-N junction nanowires. J. Am. Chem. Soc..

[34] Park S., Lim J.H., Chung S.W., Mirkin C.A. (2004). Self-assembly of mesoscopic metal-polymer amphiphiles. Science.

[35] Lin H.W., Liu H.B., Qian X.M., Lai S.W., Li Y.J., Chen N., Ouyang C.B., Che C.M., Li Y.L. (2011). Constructing a blue light photodetector on inorganic/organic p-n heterojunction nanowire arrays. Inorg. Chem..

[36] Xie Y.B., Du H.X. (2012). Electrochemical capacitance performance of polypyrrole-titania nanotube hybrid. J. Solid State Electr..

[37] Butterworth M.D., Bell S.A., Armes S.P., Simpson A.W. (1996). Synthesis and characterization of polypyrrole-magnetite-silica particles. J. Colloid Interface Sci..

[38] Shin S., Yoon H., Jang J. (2008). Polymer-encapsulated iron oxide nanoparticles as highly efficient Fenton catalysts. Catal. Commun..

[39] Zhang X.Y., Goux W.J., Manohar S.K. (2004). Synthesis of polyaniline nanofibers by “nanofiber seeding”. J. Am. Chem. Soc..

[40] Zhang X.Y., Manohar S.K. (2004). Bulk synthesis of polypyrrole nanofibers by a seeding approach. J. Am. Chem. Soc..

[41] Liu Z., Poyraz S., Liu Y., Zhang X.Y. (2012). Seeding approach to noble metal decorated conducting polymer nanofiber network. Nanoscale.

[42] Kwon O.S., Park S.J., Lee J.S., Park E., Kim T., Park H.W., You S.A., Yoon H., Jang J. (2012). Multidimensional conducting polymer nanotubes for ultrasensitive chemical nerve agent sensing. Nano Lett..

[43] Yoon H., Chang M., Jang J. (2007). Formation of 1D poly(3,4-ethylenedioxythiophene) nanomaterials in reverse microemulsions and their application to chemical sensors. Adv. Funct. Mater..

[44] Wang F., Gu H.W., Swager T.M. (2008). Carbon nanotube/polythiophene chemiresistive sensors for chemical warfare agents. J. Am. Chem. Soc..

[45] Tiwari D.C., Sharma R., Vyas K.D., Boopathi M., Singh V.V., Pandey P. (2010). Electrochemical incorporation of copper phthalocyanine in conducting polypyrrole for the sensing of DMMP. Sens. Actuator B Chem..

[46] Shirsat M.D., Bangar M.A., Deshusses M.A., Myung N.V., Mulchandani A. (2009). Polyaniline nanowires-gold nanoparticles hybrid network based chemiresistive hydrogen sulfide sensor. Appl. Phys. Lett..

[47] Virji S., Fowler J.D., Baker C.O., Huang J.X., Kaner R.B., Weiller B.H. (2005). Polyaniline manofiber composites with metal salts: Chemical sensors for hydrogen sulfide. Small.

[48] Andringa A.-M.P.C., Katsouras I., Blom P.W.M., de Leeuw D.M. (2014). NO_2_ detection and real-time sensing with field-effect transistors. Chem. Mater..

[49] Geng L.N., Huang X.L., Zhao Y.Q., Li P., Wang S.R., Zhang S.M., Wu S.H. (2006). H_2_S sensitivity study of polypyrrole/WO_3_ materials. Solid State Electron..

[50] Geng L., Wang S.R., Zhao Y.Q., Li P., Zhang S.M., Huang W.P., Wu S.H. (2006). Study of the primary sensitivity of polypyrrole/r-Fe_2_O_3_ to toxic gases. Mater. Chem. Phys..

[51] Zhang D.F., Sun L.D., Xu G., Yan C.H. (2006). Size-controllable one-dimensinal SnO_2_ nanocrystals: Synthesis, growth mechanism, and gas sensing property. Phys. Chem. Chem. Phys..

[52] Jimenez I., Arbiol J., Dezanneau G., Cornet A., Morante J.R. (2003). Crystalline structure, defects and gas sensor response to NO_2_ and H_2_S of tungsten trioxide nanopowders. Sens. Actuator B Chem..

[53] Fan F.Y., Feng Y.J., Bai S.L., Feng J.T., Chen A.F., Li D.Q. (2013). Synthesis and gas sensing properties to NO_2_ of ZnO nanoparticles. Sens. Actuator B Chem..

[54] Kong F.H., Wang Y., Zhang J., Xia H.J., Zhu B.L., Wang Y.M., Wang S.R., Wu S.H. (2008). The preparation and gas sensitivity study of polythiophene/SnO_2_ composites. Mater. Sci. Eng. B Adv..

[55] Huang J., Kang Y.F., Yang T.L., Wang Y., Wang S.R. (2011). Preparation of polythiophene/WO_3_ organic-inorganic hybrids and their gas sensing properties for NO_2_ detection at low temperature. J. Natl. Gas. Chem..

[56] Sadek A.Z., Wlodarski W., Shin K., Kaner R.B., Kalantar-zadeh K. (2006). A layered surface acoustic wave gas sensor based on a polyaniline/In_2_O_3_ nanofibre composite. Nanotechnology.

[57] Yang X.M., Li L.A., Zhao Y. (2010). Ag/AgCl-decorated polypyrrole nanotubes and their sensory properties. Synth. Met..

[58] Hong L.J., Li Y., Yang M.J. (2010). Fabrication and ammonia gas sensing of palladium/polypyrrole nanocomposite. Sens. Actuator B Chem..

[59] Park E., Kwon O.S., Park S.J., Lee J.S., You S., Jang J. (2012). One-pot synthesis of silver nanoparticles decorated poly(3,4-ethylenedioxythiophene) nanotubes for chemical sensor application. J. Mater. Chem..

[60] Gong J., Li Y.H., Hu Z.S., Zhou Z.Z., Deng Y.L. (2010). Ultrasensitive NH_3_ gas sensor from polyaniline nanograin enchased TiO_2_ fibers. J. Phys. Chem. C.

[61] Kwon O.S., Park S.J., Yoon H., Jang J. (2012). Highly sensitive and selective chemiresistive sensors based on multidimensional polypyrrole nanotubes. Chem. Commun..

[62] Phillips M., Gleeson K., Hughes J.M.B., Greenberg J., Cataneo R.N., Baker L., McVay W.P. (1999). Volatile organic compounds in breath as markers of lung cancer: A cross-sectional study. Lancet.

[63] Athawale A.A., Bhagwat S.V., Katre P.P. (2006). Nanocomposite of Pd-polyaniline as a selective methanol sensor. Sens. Actuator B Chem..

[64] Choudhury A. (2009). Polyaniline/silver nanocomposites: Dielectric properties and ethanol vapour sensitivity. Sens. Actuator B Chem..

[65] Xian Y.Z., Hu Y., Liu F., Xian Y., Wang H.T., Jin L.T. (2006). Glucose biosensor based on Au nanoparticles-conductive polyaniline nanocomposite. Biosens. Bioelectron..

[66] Xu L.H., Zhu Y.H., Yang X.L., Li C.Z. (2009). Amperometric biosensor based on carbon nanotubes coated with polyaniline/dendrimer-encapsulated Pt nanoparticles for glucose detection. Mater. Sci. Eng. C Bio S.

[67] Liu Z., Wang J., Xie D.H., Chen G. (2008). Polyaniline-coated Fe_3_O_4_ nanoparticle-carbon-nanotube composite and its application in electrochemical biosensing. Small.

[68] Guo S.J., Dong S.J., Wang E.K. (2009). Polyaniline/Pt hybrid nanofibers: High-efficiency nanoelectrocatalysts for electrochemical devices. Small.

[69] Bian X.J., Lu X.F., Jin E., Kong L.R., Zhang W.J., Wang C. (2010). Fabrication of Pt/polypyrrole hybrid hollow microspheres and their application in electrochemical biosensing towards hydrogen peroxide. Talanta.

[70] Li F., Yang L.M., Zhao C., Du Z.F. (2011). Electroactive gold nanoparticles/polyaniline/polydopamine hybrid composite in neutral solution as high-performance sensing platform. Anal. Methods.

[71] Qian T.Y.C., Zhou X., Wu S., Shen J. (2014). Au nanoparticles decorated polypyrrole/reduced graphene oxide hybrid sheets for ultrasensitive dopamine detection. Sens. Actuator B Chem..

[72] Tian S.J., Liu J.Y., Zhu T., Knoll W. (2004). Polyaniline/gold nanoparticle multilayer films: Assembly, properties, and biological applications. Chem. Mater..

